# Genomic Epidemiology of SARS-CoV-2 in Ukraine from May 2022 to March 2024 Reveals Omicron Variant Dynamics

**DOI:** 10.3390/v17071000

**Published:** 2025-07-17

**Authors:** Anna Iaruchyk, Jason Farlow, Artem Skrypnyk, Serhii Matchyshyn, Alina Kovalchuk, Iryna Demchyshyna, Mykhailo Rosada, Aron Kassahun Aregay, Jarno Habicht

**Affiliations:** 1 World Health Organization Country Office, 01021 Kyiv, Ukrainematchyshyns@who.int (S.M.);; 2 Pathogen and Microbiome Institute, Northern Arizona University, Flagstaff, AZ 86011, USA; 3 Public Health Center of the Ministry of Health of Ukraine, 04071 Kyiv, Ukraine

**Keywords:** SARS-CoV-2, genomic epidemiology, genomic surveillance, Omicron, whole genome sequencing, variant dynamics, COVID-19 surveillance, SARS-CoV-2 introductions, pandemic, public health

## Abstract

In Ukraine, SARS-CoV-2 detection and national genomic surveillance have been complicated by full-scale war, limited resources, and varying levels of public health infrastructure impacted across the country. Following the Spring of 2022, only a paucity of data have been reported describing the prevalence and variant dynamics of SARS-CoV-2 in the country. Comparative whole genome analysis has overtaken diagnostics as the new gold standard for detecting and tracing emerging variants while showing utility to rapidly inform diagnostics, vaccine strategies, and health policy. Herein, we provide an updated report characterizing the dynamics and prevalence of SARS-CoV-2 in Ukraine from 1 May 2022 to 31 March 2024. The present study extends previous reports for disease incidence Waves 1–4 in Ukraine with the addition herein of Waves 5, 6, and 7, occurring from August to November 2022 (Wave 5), February to May 2023 (Wave 6), and October 2023 to January 2024 (Wave 7). During the study period, the national Case Fatality Rate (CFR) fluctuated between 0.46% and 1.74%, indicating a consistent yet modest rate when compared to the global average. The epidemiological dynamics of Variants of Concern (VOCs) in Ukraine reflected global patterns over this period, punctuated by the rise of the BA.5 lineage and its subsequent replacement by the Omicron subvariants XBB and JN.1. Our analysis of variant dispersal patterns revealed multiple potential spatiotemporal introductions into Ukraine from Europe, Asia, and North America. Our results highlight the importance of ongoing genomic surveillance to monitor variant dynamics and support global efforts to control and mitigate COVID-19 disease risks as new variants arise.

## 1. Introduction

The severe acute respiratory syndrome coronavirus 2 (SARS-CoV-2), causing COVID-19, was first detected in December 2019 in Wuhan, Hubei, China [1,2]. The virus spread rapidly worldwide, and the World Health Organization (WHO) officially declared a pandemic on 11 March 2020 [3]. By the end of 2023, more than 772 million confirmed cases of COVID-19 and nearly seven million deaths had been reported globally [4].

SARS-CoV-2 representatives with elevated transmission rates compared to previous variants, termed ‘variants of concern’ (VOCs), are grouped relative to their lineage and component mutations [5]. Since its emergence in 2019, SARS-CoV-2 has evolved into multiple VOCs, changing the pandemic’s dynamics from Alpha to Beta, Gamma, Delta, and Omicron [6]. Global genomic surveillance utilizing next-generation sequencing (NGS) has proven essential for tracking the evolution and spread of SARS-CoV-2 VOCs and their subvariants, providing critical and timely high-resolution phylodynamic characterization [7,8]. The rapid growth of available genomic data through international networks like GISAID has proven invaluable for monitoring viral dynamics and making informed decisions to manage the pandemic effectively [9,10]. Despite high infection rates in 2022, case severity decreased, likely due to vaccination and prior infections, improved health management, and the reduced virulence of Omicron sublineages [11,12]. These factors led to a significant decline in COVID-19 deaths and hospitalizations, prompting the WHO to declare the end of the emergency phase in May 2023 [13]. However, the WHO continues to coordinate the global response and track new SARS-CoV-2 as the virus continues to evolve.

In Ukraine, the public health response to the SARS-CoV-2 pandemic has been complicated by various factors, including limited resources, full-scale war, and varying levels of public health infrastructure impacted across the country. The first case of COVID-19 was confirmed in Ukraine on 3 March 2020 in a person who recently visited Italy [14]. Subsequently, approximately 5.5 million cases and 112,000 deaths have been reported in the country during the pandemic, according to the Ministry of Health of Ukraine [15]. Similar to trends observed in other countries, the course of the epidemic in Ukraine follows distinct wave-like spatiotemporal patterns in case frequency and variant prevalence [16,17]. Previously, Yakovleva et al. reported four periods of peak COVID-19 incidence comprising Wave 1 (1 September 2020–31 January 2021), Wave 2 (1 February 2021–9 June 2021), Wave 3 (10 June 2021 to 31 December 2021), and Wave 4 (1 January–25 April 2022) [16]. Wave 3, dominated by the Delta VOC, was introduced into Ukraine in the summer, with most inferred introductions from central and eastern European countries [16]. In early January 2022, the Omicron VOC was first detected in Ukraine and quickly overtook Delta, becoming the dominant variant in the following epidemiological wave [17].

In the present study, we extend the findings of Yakovleva et al. and Gerashchenko et al. and investigate the dynamics and prevalence of SARS-CoV-2 in Ukraine from 1 May 2022 to 31 March 2024. Our data revealed patterns in the dispersal and duration of the Omicron VOC and select subvariants monitored through active genomic surveillance, as well as spatiotemporal introductions from Europe, North America, and Asia. Our results highlight the importance of ongoing genomic surveillance to monitor variant dynamics and support international efforts to control and mitigate COVID-19 disease risks as new variants arise.

## 2. Materials and Methods

### 2.1. Genomic Data Acquisition

The metadata and nucleotide sequences of SARS-CoV-2 samples were extracted from the GISAID database [9,10] via the download tool in the EpiCoV section with the following filters: Location “Europe/Ukraine/…”, host “Human”, samples associated with a complete collection date (year-month-day) spanning the period from 1 May 2022 to 31 March 2024, submission date before 31 May 2024, “Low coverage excluded”. This initial dataset included 3995 submitted samples (Table S2). In the next step, we analyzed the initial dataset for sequence quality check using the web server version of Nextclade [18,19]. According to the Nextclade analysis results, 397 sequences out of 3995 have “bad” QC overall status, 1017—“mediocre”, and 2581—“good” QC overall status. Sequences designated as “Bad” also included 4 low-quality Delta genomes. We filtered out 397 sequences with a “bad” QC overall status from our dataset and used the remaining 3598 sequences for further lineage distribution and phylogenetic analyses.

Our dataset includes samples from all available oblasts of Ukraine over the study period (Table S3). Genomic data from Luhansk and Donetsk oblasts and Crimea were not incorporated due to the occupation by Russia and ongoing military actions in those areas.

### 2.2. Epidemiological Analysis

Epidemiological data for COVID-19 cases, hospitalizations, and case fatality were collected through the national epidemiological surveillance system and retrieved from the Public Health Center of the Ministry of Health of Ukraine (UPHC). The dataset presents two notable limitations: (1) data exclusion from territories occupied by the Russian Federation (including Crimea, Sevastopol, Luhanska oblast, and portions of Donetska, Zaporizka, and Khersonska oblasts) and (2) missing data for weeks 27–40 of 2023 (3 July 2023–1 October 2023).

Pearson correlation coefficients were calculated to assess the linear relationships between COVID-19 variant prevalence and severity metrics (hospitalization rate and case fatality rate). Only complete cases were used in the analysis to ensure all correlations were based on the same time periods. Statistical analysis was performed using R software (version 4.3.0) with the corrplot package for visualization.

### 2.3. VOC Lineage Distribution

The monthly lineage distribution of 3598 samples was analyzed using metadata downloaded from GISAID. Calculations in percentage relative to the total number of samples were determined for the following lineages per PANGO nomenclature: BA.1; BA.2, excluding BA.2.75, BA.2.86, BA.2.75; BA.2.86 excluding JN.1, JN.1.; JN.1; BA.4; BA.5; XBB excluding XBB.1.5, EG.5; XBB.1.5; EG.5; and other recombinants (Supplementary Table S1). The reported lineages were selected based on the VOC/VOI status designated by the WHO based on their assessed potential (1) for expansion and replacement of prior variants, (2) for causing new waves with increased circulation, and (3) for the need for adjustments to public health actions [20].

### 2.4. Sequencing

Ukraine submitted a total of 3957 Ukrainian SARS-CoV-2 samples to the GISAID database that were used in this study and sequenced by the Reference Laboratory for Diagnostics of HIV/AIDS, Virological and Especially Dangerous Pathogens of the Public Health Center of the Ministry of Health of Ukraine (Virology Laboratory of UPHC). In addition, we incorporated 38 Ukrainian samples sequenced by CNR Virus des Infections Respiratoires, France SUD. Library preparation for genomes submitted previously by UPHC utilized the Ion Ampliseq SARS-CoV-2 Research Panel and SARS-CoV-2 Insight Research Assay Panel (ThermoFisher, Waltham, MA, USA) and sequencing on the Ion GeneStudio™ S5 instrument (Thermo Fisher Scientific, Waltham, MA, USA).

### 2.5. Phylogenetics

Viral genome alignment, mutation calling, Pango lineage assignment, clade assignment, and phylogenetic placement were determined online using the default greedy parsimony tree algorithm and associated tools of the Nextclade webserver v3.1.0 (https://clades.nextstrain.org/ accessed on 20 September 2024) (19) and the Pangolin (Phylogenetic Assignment of Named Global Outbreak Lineages) Webserver v4.3 (https://pangolin.cog-uk.io/ accessed on 20 September 2024). The Wuhan-Hu-1/2019 (MN908947) reference genome was used by the Nextclade server.

## 3. Results

### 3.1. Dynamics of COVID-19 Cases and SARS-CoV-2 VOCs in Ukraine

**Figure 1:**
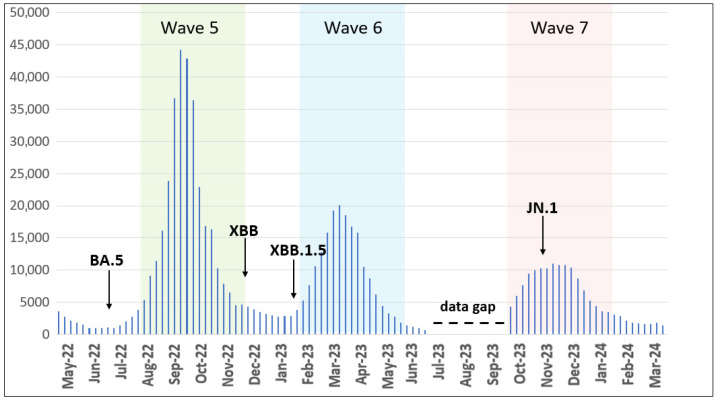
Epidemic curve of confirmed COVID-19 cases registered in Ukraine from May 2022 to March 2024. Waves 5–7 are highlighted by colors. Arrows show the first detected cases of the indicated VOC/VOI. The data gap is elaborated in the discussion section. The number of cases is plotted along the y-axis.

We analyzed retrieved epidemiological data for COVID-19 cases between May 2022 and March 2024 and observed the following distinct epidemic waves in Ukraine. Wave 5 occurred from August to November 2022, Wave 6 from February to May 2023, and Wave 7 from October 2023 to January 2024 (Figure 1).

**Figure 2:**
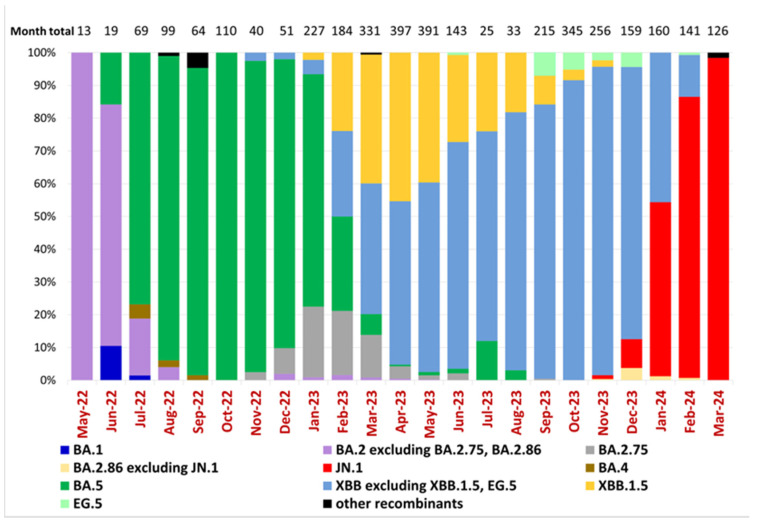
Variant frequencies of SARS-CoV-2 samples collected in Ukraine in May 2022–March 2024. The graph shows the monthly variant frequencies as a percentage relative to the total number of sequenced samples per month. Variants per PANGO nomenclature are represented by distinct colors, as indicated in the legend. Sample collection dates are plotted along the x-axis. The percentage of variant frequency relative to the total number of samples per month is plotted along the y-axis. The total number of sequenced samples for each month is shown above the bars (“Month total”).

According to a previous study of Ukrainian SARS-CoV-2 sequences [16], the Omicron BA.1-related sub-lineage was first identified on 19 December 2021 and then predominated from January to February 2022, while the first confirmed BA.2 sublineage sequence was reported on 19 January 2022. Epidemiological dynamics in Ukraine over the current study period reflected the predominance of five Omicron lineages and subvariants starting with BA.2, the subsequent emergence of BA.5, and its replacement by Omicron subvariants XBB and finally JN.1 (Figure 2). Our dataset, beginning in May 2022, shows that the BA.2 sublineage predominated in Ukraine until July 2022, when it was replaced by the BA.5 sublineage, which remained dominant until February 2023 (Figure 2). In Ukraine, the first cases of BA.2.75 and XBB were detected in November 2022 (23 November 2022 and 28 November 2022, respectively), and the first case of XBB.1.5 was identified on 21 January 2023. From March to December 2023, the XBB sublineage, including XBB.1.5 and EG.5, predominated in Ukraine. Other XBB subtypes overtook XBB.1.5 beginning in May of 2023. In November 2023, the JN.1 sublineage was detected in Ukraine and remained the dominant circulating variant (Figure 2).

### 3.2. Evolutionary Relationships of SARS-CoV-2 in Ukraine

**Figure 3:**
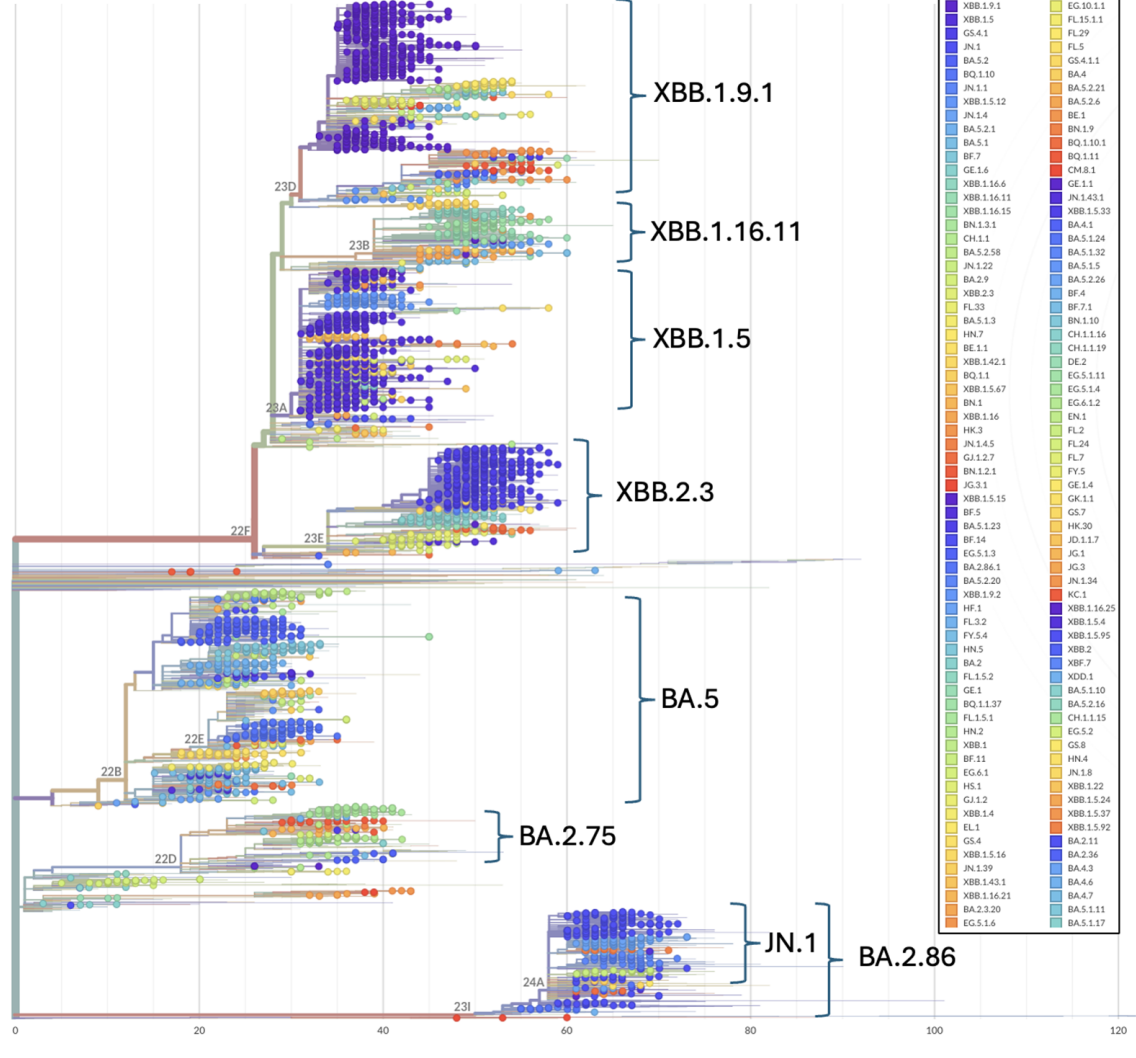
Phylogenetic relationships among Omicron SARS-CoV-2 genomes included in this study. Phylogenetic reconstructions and clade assignment were performed using the Nextclade web server v3.1.0 under default settings.

Phylogenetic patterns over the study period illustrate the topological placement of the BA.2 Omicron lineages dominating Wave 4 of the pandemic in Ukraine (Figure 2 and Figure 3). Phylogenetic relationships also depict the highly diverse Omicron subvariants BA.5 (Wave 5), the three major recombinant XBB lineages (Wave 6) that convergently acquired the R346T, N460K, and F486S mutations (Figure 2 and Figure 3) and the divergent clade comprising JN.1 and BA.2.86 representatives (Figure 3).

### 3.3. Introduction of Omicron Variants into Ukraine

**Figure 4:**
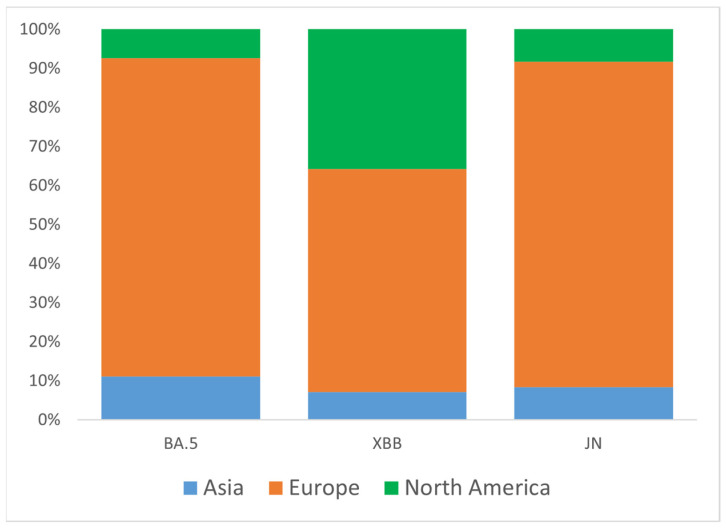
Proportions of inferred SARS-CoV-2 introductions into Ukraine by continent of origin, stratified by major clade (BA.5, XBB, JN). The percentage of introductions from different continents relative to the total number of introductions within each clade is plotted along the y-axis.

To better understand the origin of Omicron introductions into Ukraine, we analyzed three major clades (groups) of VOC circulated during the study period: BA.5, XBB, and JN. Representative sets of Ukrainian samples from each of the three major groups were randomly down-sampled to N = 100 and analyzed for global genetic relationships using the GISAID Audacity Instant tool [21]. Based on near relative phylogenetic placement from Audacity output, we identified potential introduction events for BA.5 (N = 27), XBB (N = 14), and JN (N = 12). As expected, most candidate introductions were from European countries (Austria, Belgium, Bulgaria, Czech Republic, Denmark, England, Finland, France, Germany, Greece, Moldova, Poland, Romania, Slovakia, Spain, Sweden, Switzerland), as well as from the USA and Asia (Israel, Oman, South Korea) (Figure 4). For the XBB variant, the portion of introductions from the USA was higher than for the BA.5 and JN variants, reaching 36% (Figure 4).

### 3.4. Confirmed COVID-19 Cases by Oblast

**Figure 5:**
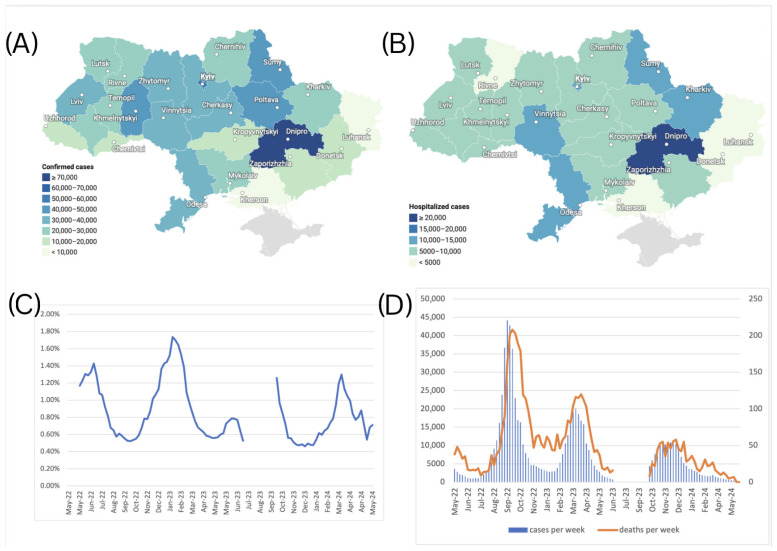
(**A**) Number of confirmed cases of COVID-19 by oblasts of Ukraine, May 2022–March 2024. (**B**) Number of hospitalizations due to COVID-19 by oblasts of Ukraine, May 2022–March 2024. (**C**) Case Fatality Rate (CFR) for COVID-19 in Ukraine from May 2022 to March 2024, calculated using a 5-week moving average for cases and deaths, with an applied 2-week lag between case and death registration. (**D**) Scaled number of death cases per week, plotted against the number of confirmed cases per week (left axis for confirmed cases, right axis for death cases). The graph for death cases was magnified using a different scale to make the variability visible.

A total of 712,072 confirmed cases of COVID-19 were reported in Ukraine during May 2022–March 2024. Among others, the Dnipropetrovsk oblast reported the highest number of cases at 73,291 (10.3% of the total), followed by Kyiv city with 54,838 cases (7.7%) (Figure 5A), which is consistent with the current dense population in these areas. Central and northern regions, such as Kyiv and Zhytomyr oblasts, reported moderate numbers, with 36,288 cases (5.1%) and 36,050 cases (5.1%), respectively. In contrast, frontline regions like Luhansk, Kherson, and Donetsk oblasts reported substantially fewer confirmed cases due to war-related disruptions. The Luhansk oblast recorded only 3 cases (0.0%), while Kherson and Donetsk oblasts reported 1917 cases (0.3%) and 14,511 cases (2.0%), respectively. Due to the Russian occupation, no health records were submitted to Ukraine from the Luhansk oblast after February 2022. Western oblasts such as Lviv and Khmelnytskyi reported 34,083 cases (4.8%) and 44,406 cases (6.2%), respectively, reflecting the impact of internally displaced populations and comparatively more stable healthcare systems (Figure 5A).

### 3.5. Hospitalization Cases by Oblast

A total of 198,875 hospitalizations due to COVID-19 were reported in Ukraine over the study period. The regional distribution of respective hospitalizations over the study period is shown in Figure 5B. Similar to the observed confirmed COVID-19 case trends, the Dnipropetrovsk oblast recorded the highest number of hospitalizations, with 23,350 cases (11.7% of the total), followed by Kyiv city with 13,880 hospitalizations (7.0%). In contrast, oblasts closer to the frontline, such as Kherson, reported minimal hospitalizations—1250 cases (0.6%). Similarly, Donetsk reported 4099 cases (2.1%), and Zaporizhzhia recorded 5054 cases (2.5%), both showing reduced hospitalization numbers likely impacted by limited healthcare access in conflict-affected areas. Western regions such as Lviv, Ivano-Frankivsk, and Zakarpattia reported moderate hospitalization shares, with 9464 cases (4.8%), 9223 cases (4.6%), and 5318 cases (2.7%), respectively, likely driven by the influx of internally displaced persons fleeing from conflict zones. Central regions, including Vinnytsia and Khmelnytskyi, also recorded higher hospitalization figures, with 13,646 cases (6.9%) in Vinnytsia and 8066 cases (4.1%) in Khmelnytskyi, respectively. Hospitalization rates also illustrate oblast-specific differences in clinical admission criteria, as some oblasts reported four times higher hospitalization rates per 100 cases when compared to other oblasts.

### 3.6. Case Fatality Rates

The Case Fatality Rate (CFR) dynamics in Ukraine during the study period are illustrated in Figure 5C. During the study period, the Case Fatality Rate (CFR) fluctuated between 0.46% and 1.74%, when calculated with a 5-week moving average applied to case and death counts to reduce reporting noise, and a 2-week lag applied to account for the average natural lag between the case registration and death registration, seen in Figure 5D. This indicates that Ukraine’s CFR displayed a consistent yet modest increase above the global average during the study period [22], suggesting elevated COVID-19 mortality risk. Several factors may have contributed to this elevated CFR, including healthcare system capacity constraints due to the ongoing war, demographic shifts resulting from the wartime population displacement of younger cohorts, and the consequent relative increase in vulnerable populations with higher baseline mortality risk.

### 3.7. VOCs and Disease Severity

**Figure 6:**
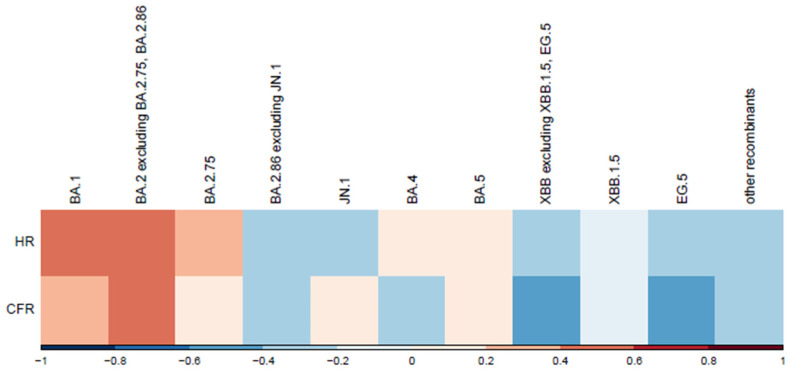
Correlation between variants and normalized severity metrics. This heatmap illustrates the correlation between SARS-CoV-2 variants and two normalized severity metrics: hospitalization rate (HR) and Case Fatality Rate (CFR) per 100 cases. SARS-CoV-2 variants are displayed chronologically. Correlation coefficients are represented along the x-axis, with red indicating positive correlations (stronger associations with increased severity) and blue showing negative correlations (weaker associations or reduced severity).

Correlations between variants and normalized disease severity metrics are shown in Figure 6. We observed a moderate positive correlation for variants BA.1 and BA.2, excluding BA.2.75, BA.2.86. A moderate negative correlation was observed for variants EG.5 and XBB, excluding XBB.1.5. Hospitalization and death counts in our analysis served as proxies for disease severity. To mitigate potential bias from absolute number increases, the correlation was analyzed between variant spread percentages and normalized severity metrics (hospitalization and case fatality rates per 100 cases). A more in-depth analysis is warranted to account for other factors, such as vaccination rate. Lower vaccination rates during earlier epidemic stages likely contributed to the higher hospitalization and case fatality rates observed for the first variants in the correlation plot (Figure 6).

## 4. Discussion

In the present study, we characterized the dynamics and prevalence of SARS-CoV-2 in Ukraine from 1 May 2022 to 31 March 2024. The present study extends previous reports for disease incidence Waves 1–4 in Ukraine with the addition here of Waves 5, 6, and 7 occurring from August to November 2022 (Wave 5), February to May 2023 (Wave 6), and October 2023 to January 2024 (Wave 7) (Figure 1). Our data reflect similar published reports [23,24] that illustrate the emergence and dominance of BA.5 in the summer of 2022, transition to XBB relatives in the spring of 2023, and the subsequent rise of JN.1 encoding the hallmark Leu455Ser mutation in the winter of 2024.

Variant predominance over the cocirculation period occurred over a 10-month period. Epidemiological dynamics in Ukraine reflected global patterns punctuated by the rise of the BA5 lineage and its subsequent replacement by Omicron subvariants XBB and JN.1.

Comparing variant dynamics with national COVID-19 waves, we observed that the start of each wave during our study period coincided with the emergence of a new variant of concern/interest in Ukraine (Figure 1 and Figure 2). This underscores the critical role of continuous genomic surveillance in detecting new SARS-CoV-2 variants promptly, enabling informed public health interventions to mitigate the impact of future waves.

### 4.1. Epidemiological Data Collection

Following the WHO statement on 5 May 2023, declaring COVID-19 an established and ongoing health issue that no longer constitutes a public health emergency of international concern [13], Ukraine officially ended the national quarantine on 27 June 2023. Subsequently, changes were introduced to the national COVID-19-related data collection. During the national quarantine, detailed epidemiological data were collected for individual COVID-19 cases using an Electronic Integrated Disease Surveillance System (EIDSS). After the cancellation of quarantine restrictions in mid-2023, only aggregated counts of COVID-19 cases, hospitalizations, and deaths were collected through the national routine surveillance system for respiratory diseases. As this surveillance system functions only during the epidemic season between the 40th week of the current year and the 20th week of the following year, the epidemiological data reported here contain a gap corresponding to weeks 27–40 of 2023 (3 July 2023–1 October 2023, Figure 1).

To align with monthly genomic surveillance data, weekly data were aggregated by calendar month. The COVID-19 hospitalization rate was calculated by dividing all cases of hospitalization by all confirmed cases for that month. Similarly, the Case Fatality Rate was calculated by dividing all cases of death by all confirmed COVID-19 cases for that month.

### 4.2. Impact of Russian Invasion During the COVID-19 Pandemic

Ukraine, like other countries, took public health measures to control the pandemic yet faced a pronounced challenge due to the full-scale invasion by Russia in February 2022 during the height of the pandemic. This military aggression led to large displacement of the Ukrainian population, caused disruptions in COVID-19 testing and the implementation of immunization programs, and limited broad access to essential health care and emergency response systems. For example, as of 27 June 2023, only 15.5 million people, which is 38% of the total permanent population of Ukraine, had received two doses of the vaccine [25]. The war also limited the availability of health data in the temporarily occupied territories, resulting in incomplete statistics [26,27]. The Dnipropetrovsk oblast displayed the highest number of COVID-19 cases and hospitalizations over the study period (Figure 5A,B). This trend may reflect the influence of population movements due to the ongoing war in Ukraine, as the Dnipropetrovsk oblast became a hub for internally displaced people from neighboring oblasts. In 2024, approximately 3.6 million internally displaced persons were recorded in Ukraine by the International Organization for Migration [28], a factor that may have contributed to increased population density and transmission rates.

Disease control efforts continue to be hampered by limited resources and sustained assaults on health facilities throughout the country [29]. While COVID-19 case positivity, associated emergency response visits, and COVID-related hospitalizations have declined, the Virology Laboratory of the Public Health Center continues genomic surveillance and VOC monitoring. Our results highlight the critical importance of ongoing genomic and epidemiological surveillance to monitor both variant and disease dynamics as new variants emerge. Despite significant challenges, Ukraine’s public health sector has continued epidemiological data collection, SARS-CoV-2 sequencing, and analysis, demonstrating the resilience of its surveillance systems and the need to sustain essential public health functions in humanitarian settings. Throughout the COVID-19 pandemic, substantial investments were made in the laboratory capacities of Ukraine’s public health system, including sequencing capabilities, with support from the WHO and other international partners. These efforts, initiated before the full-scale invasion and sustained throughout the study period, have enabled SARS-CoV-2 genomic surveillance in Ukraine and contributions to global variant monitoring. Given the ongoing war and the emergence of new variants, maintaining and expanding these capacities remains essential for public health preparedness and response.

## Data Availability

The SARS-CoV-2 genomes analyzed in this study are available on the Global Initiative on sharing Avian Influenza Data (GISAID) database (https://gisaid.org/, accessed on 15 November 2024) and were listed in Table S2.

## Supplementary Materials

The following supporting information can be downloaded at https://www.mdpi.com/article/10.3390/v17071000/s1: Table S1: Monthly lineage distribution of 3598 Ukrainian SARS-CoV-2 samples downloaded from GISAID and used in this study. Table S2: GISAID data availability. Table S3: Geographic representativeness of Ukrainian SARS-CoV-2 genomes.

## Abbreviations

The following abbreviations are used in this manuscript:

CFR	Case Fatality Rate
COVID-19	Coronavirus Disease 2019
EIDSS	Electronic Integrated Disease Surveillance System
GISAID	Global Initiative on Sharing All Influenza Data
HIV/AIDS	Human Immunodeficiency Virus/Acquired Immunodeficiency Syndrome
HR	Hospitalization Rate
NGS	Next Generation Sequencing
PANGO	Phylogenetic Assignment of Named Global Outbreak Lineages
QC	Quality Control
SARS-CoV-2	Severe Acute Respiratory Syndrome Coronavirus 2
UPHC	Public Health Center of the Ministry of Health of Ukraine
VOC	Variant of Concern
VOI	Variant of Interest
WHO	World Health Organization
